# Validation of the Spanish version of the Chronic Pain Acceptance Questionnaire (CPAQ) for the assessment of acceptance in fibromyalgia

**DOI:** 10.1186/1477-7525-8-37

**Published:** 2010-04-12

**Authors:** Baltasar Rodero, Javier García-Campayo, Benigno Casanueva, Yolanda Lopez del Hoyo, Antoni Serrano-Blanco, Juan V Luciano

**Affiliations:** 1 Department of Psychology, Centro Rodero, Clínica de Neurociencias, Santander, Spain; 2 Department of Psychiatry, Miguel Servet University Hospital, Instituto Aragonés de Ciencias de la Salud, Spain; 3 Rheumatology Clinic, Santander, Spain; 4 Department of Psychology, University of Zaragoza, Instituto Aragonés de Ciencias de la Salud, Spain; 5 Research and Development Unit - Parque Sanitario Sant Joan de Déu & Fundación Sant Joan de Déu, Sant Boi de Llobregat, Barcelona, Spain, REDIAPP "Red de Investigación en Actividades Preventivas y Promoción de la Salud" (Research Network on Preventative Activities and Health Promotion) (RD06/0018/0017; 6 Instituto Aragonés de Ciencias de la Salud, Zaragoza, Spain

## Abstract

**Background:**

The aim of this study was to validate a Spanish version of the Chronic Pain Acceptance Questionnaire (CPAQ). Pain acceptance is the process of giving up the struggle with pain and learning to live a worthwhile life despite it. The Chronic Pain Acceptance Questionnaire (CPAQ) is the questionnaire most often used to measure pain acceptance in chronic pain populations.

**Methods:**

A total of 205 Spanish patients diagnosed with fibromyalgia syndrome who attended our pain clinic were asked to complete a battery of psychometric instruments: the Pain Visual Analogue Scale (PVAS) for pain intensity, the Hospital Anxiety and Depression Scale (HADS), the Medical Outcome Study Short Form 36 (SF-36), the Pain Catastrophising Scale (PCS) and the Fibromyalgia Impact Questionnaire (FIQ).

**Results:**

Analysis of results showed that the Spanish CPAQ had good test-retest reliability (intraclass correlation coefficient 0.83) and internal consistency reliability (Cronbach's α: 0.83). The Spanish CPAQ score significantly correlated with pain intensity, anxiety, depression, pain catastrophising, health status and physical and psychosocial disability. The Scree plot and a Principal Components Factor analysis confirmed the same two-factor construct as the original English CPAQ.

**Conclusion:**

The Spanish CPAQ is a reliable clinical assessment tool with valid construct validity for the acceptance measurement among a sample of Spanish fibromyalgia patients. This study will make it easier to assess pain acceptance in Spanish populations with fibromyalgia.

## Background

Fibromyalgia is a chronic musculoskeletal pain disorder of unknown aetiology, characterised by widespread pain and muscle tenderness and often accompanied by fatigue, sleep disturbance and depressed mood [1,2]. The prognosis for symptomatic recovery is generally poor, and the estimation for lifetime prevalence is approximately 2% in community samples [3]. The syndrome's pathology is not well understood and, to date, no treatment has proven effective in fully alleviating its symptoms.

In the last decade, "acceptance" has emerged as a valuable construct for contextual or third wave psychotherapies. Although sometimes misinterpreted as surrender [4], the real concept is far from this idea. Acceptance of chronic pain involves the individual reducing unsuccessful attempts to avoid or control pain and focusing instead on participation in valued activities and the pursuit of personally relevant goals [5].

Hayes described, for the first time, a general measure of acceptance and experiential avoidance, the Acceptance and Action Questionnaire [6,7]. The CPAQ was developed by Geiser [8] as an adaptation of this assessment for patients with chronic pain. Subsequent analyses carried out by McCracken [5,9,10] critically evaluated the content and factor structure, improving the questionnaire.

Factor analysis of the main tool for measuring acceptance, the Chronic Pain Acceptance Questionnaire (CPAQ), initially reveals 34 items and four components, which are as follows: activity engagement; pain willingness; thought control and chronicity. Based on evaluation of the psychometric properties of these four subscales, however, McCracken et al. [5] reduced the CPAQ to only 20 items and two subscales (activity engagement and pain willingness). A recent confirmatory factor analysis has provided further support for these 20 items and two-factor construct of the CPAQ [11].

The Activity Engagement subscale consists of eleven items and gauges the extent to which a person follows their activities in a normal way regardless of their experience of pain. The Pain Willingness subscale has nine items and measures how much a patient believes that avoiding or controlling pain are strategies that work for him. A total score is reached by combining both subscales. Previous research studies [5,9,12,13] show that acceptance of pain and willingness to act in its presence are associated with reports of lower pain intensity, less pain-related anxiety and avoidance, less depression, less physical and psychosocial disability, more daily uptime and better work status. Contrary to what is expected, pain acceptance does not correlate with pain intensity. The reason for this lack of correlation is that acceptance can be considered as similar to a personality trait, with a normal distribution in the population, and is independent from pain level. Finally, acceptance of pain predicts better adjustment on measures of patient functioning than perceived pain intensity does, which continues to be true even when pain intensity is factored out (see [14,15] for review papers on this subject).

These results imply the potential of an improved outcome for acceptance-based clinical methods for chronic pain management. The CPAQ has already been validated in German [16] and Chinese [17]; however, currently, a measure of acceptance of pain is not available in Spanish. Therefore, we translated the revised version of the CPAQ into Spanish and tested its reliability and validity in Spanish patients suffering from fibromyalgia.

## Materials and methods

### Participants

Sample size was calculated according to the recommended 10:1 ratio of the number of subjects to the number of test items [18]. The final study sample consisted of 205 patients attending the Pain Clinic (Santander, Spain) and Fibromyalgia Unit (Hospital Miguel Servet, Zaragoza) during the year 2009. To be included in the study, patients had to fulfil the American College of Rheumatology (ACR) criteria for primary fibromyalgia^1^, which was diagnosed by a Spanish National Health Service rheumatologist. The only exclusion criterion was a medical or psychiatric disorder that impeded the patient's ability to correctly answer the questionnaire. The study questionnaires and protocol were approved by the Ethical Committee of the regional health authority, and patients signed a consent form attesting to their willingness to participate in the study.

After consenting to the study, recruited patients were given a battery of questionnaires for completion. All patients completed these instruments on the day of the visit. These included a pain form for demographic and pain-related variables, including the translated Spanish version of the CPAQ to be validated, a Pain Visual Analogue Scale (PVAS) for pain intensity, and the validated Spanish versions of the Hospital Anxiety and Depression Scale (HADS), the Short Form 36 (SF 36), the Pain Catastrophising Score (PCS) and the Fibromyalgia Impact Questionnaire (FIQ).

### Translation of the CPAQ

Two researchers, who were aware of the objectives of the CPAQ, did the first translation into Spanish. Each researcher translated the questionnaire separately. Subsequently, two native English teachers who had no knowledge regarding the instrument carried out back-translations. Finally the two English versions were judged equivalent by a third native English teacher, [5]. Any differences between the translators were resolved by mutual agreement. Both translators and authors were present at the agreement. The authors read and write technical English and know the psychological construct to be assessed with the questionnaire well. We have followed the usual guidelines for cross-cultural adaptations [19]. The original authors accepted the questionnaire to be translated. They were sent the final version of the paper, and they agree with the results.

### Measurement tools

#### 1 Pain Visual Analogue Scale (PVAS)

The PVAS consists of a 10 cm long straight line whose tips represent the limits of pain intensity (none to unbearable). The patients estimated the pain intensity experienced on the same day between 0 and 10.

#### Chronic Pain Acceptance Questionnaire (CPAQ)

The Chronic Pain Acceptance Questionnaire (CPAQ) is a 20-item inventory designed to measure acceptance of pain. (see additional file 1: Spanish version of CPAQ) [5]. There are two principle factors measured by this questionnaire: activities engagement and pain willingness. All items are rated on a 0 (never true) to 6 (always true) scale. Nine items measuring pain willingness were reverse-keyed. Following the scoring procedure of McCracken et al. [5], a single total score was calculated based on the nine reverse-keyed items and the other eleven items measuring activities engagement. The maximum possible total score is 120, with a higher score indicating better acceptance. Complete information about the scoring calculation is given in the additional file 1: Spanish version of CPAQ.

#### Hospital Anxiety and Depression Score (HADS)

The HADS [20] is a self-report scale designed to screen for the presence of depression and anxiety disorders in medically ill patients. It is appropriate for use in both community and hospital settings and contains 14 items rated on 4-point Likert-type scale. Two subscales assessed depression and anxiety independently (HADS-Dep and HADS-Anx, respectively). It has been validated in a Spanish sample [21]. This is one of the most used questionnaires for the assessment of depression and anxiety in medical patients. We have used the cut-off point recommended in the validated Spanish version of the HADS [21], which is the same recommended by the original authors [20]: scoring 8+ on both the anxiety and depression scales. A cut off of 8 or more in HADS means suspected depression or anxiety.

#### Medical Outcome Study Short Form 36 (SF-36)

The Medical Outcome Study Short Form 36 (SF-36) is a 36-item instrument designed to measure general health status and health-related quality of life [22]. One item assesses perceived change in health status, while 35 items examine eight generic domains in both physical and mental health. The 8 domains include Physical Function (PF), Physical Role (RP), Bodily Pain (BP), General Health (GH), Vitality (VT), Social Function (SF), Emotional Role (RE) and Mental Health (MH). Scores on each subscale range from 0 to 100, with higher scores indicating better health status. The Spanish version of SF-36 has been shown to be reliable with good construct validity [23].

#### Pain Catastrophising Scale (PCS)

The PCS is a 13-item scale designed to assess the catastrophising cognitions of individuals by asking them to reflect on thoughts or feelings associated with present painful experiences [24]. The PCS can be subdivided into three subscales: rumination, magnification and helplessness. Each item is scored from 0 (not at all) to 4 (always), and scores range from 0 to 52. It has good temporal stability, internal consistency and validity. The Spanish version of the PCS has been validated by our team showing similar results to the original questionnaire [25]. Only the total score of the PCS was used in this investigation.

#### Fibromyalgia Impact Questionnaire (FIQ)

The Fibromyalgia Impact Questionnaire (FIQ) is a 10-item self-report questionnaire developed to measure the health status of fibromyalgia patients [26]. The first item focuses on the patient's ability to carry out muscular activities. In the next two items, patients are asked to circle the number of days in the past week they felt good and how often they missed work. Finally, the last seven questions (ability to work, pain, fatigue, morning tiredness, stiffness, anxiety and depression) are measured with the visual analogue scale. This instrument has a translated and validated Spanish version [27].

### Validation process

Patients diagnosed with fibromyalgia, fulfilling the criteria previously described, who attended our clinics during the year 2009 were invited to participate until the expected sample was completed. In a subsample of 64 patients, test-retest reliability for a 2-week interval was calculated. Face validity was assessed asking patients from the Spanish Association of Fibromyalgia whether they thought that the test could adequately measure their pain acceptance. Construct validity was determined by correlating the Spanish CPAQ scores to validated Spanish versions of various psychometric instruments and comparing the results with those obtained from the original English version. As the FIQ, HADS and PCS reflect health status, mood changes and emotional distress (catastrophising) in fibromyalgia patients, we anticipated that higher CPAQ scores would be associated with lower FIQ, HADS and PCS scores. For patients' general health wellbeing, including physical, emotional and social functions, the SF-36 is able to measure these domains under eight different subscales. We predicted that acceptance, as measured by the CPAQ, should positively correlate to SF-36 subscales. Exploratory factor analysis was carried out as part of the validity test.

### Statistics

Demographic data was analysed using the descriptive statistics of mean, standard deviation (SD) and range. Age and duration of pain were used as continuous variables. The remaining variables were used as dichotomous ones. The dichotomised categories and their prevalence for each variable are as follows: gender was dichotomised into male and female; marital status was grouped into married and single/separated/widowed; work status was divided into employed and unemployed and educational level was dichotomised into elementary/primary and secondary/tertiary. The CPAQ correlations were established with female, married, employed and secondary educational level.

The association between the Spanish CPAQ and demographic characteristics were evaluated using Pearson correlations. Cronbach's α coefficient was used to examine the internal consistency (ideally, α should range between 0.7-0.9) of the questionnaire. Test-retest reliability was assessed using analysis of variance intraclass correlation coefficients (ICC) [28]. ICC will range between 0 and 1, with values approaching 1 representing good reliability. Pearson correlations were also used to assess the relationship between CPAQ scores and other psychometric variables, such as pain intensity, anxiety, depression, pain catastrophising, health status and social functioning, as measured by various Spanish versions of the instruments. Finally, principle components analysis with varimax rotation was used to analyse the factorial structure of the Spanish version of CPAQ. All the variables studied showed a normal distribution. All statistical analyses were conducted using the Statistical Package for Social Science version 15.0 (SPSS 15.0) for Windows.

## Results

None of the participants were ruled out because of the exclusion criteria. The study sample consisted of 205 patients (90.7% women and 9.3% men) between the ages of 26 and 77 years (mean 50.0 years, SD: 9.7 years). Each of the subjects described themselves as being of European ethnic origin. On average, the patients had suffered from fibromyalgia for 12.1 years (range 1-55 years), and 25.2% had been granted a disability pension. Two-thirds (65.7%) of patients were unemployed, whereas 34.3% of patients were employed full- or part-time. The majority of the participants were married (73.6%), while the rest were single/separated/widowed (26.4%) individuals. Finally, most participants had an elementary-primary education (59%), while 41% had received a secondary-tertiary education.

The mean CPAQ total score was 40.9 (SD 18.5, range 5-102). This amounted to a mean item rating of 2.0, which most closely corresponds with the lower range of the 0-6 scale and the rating category "Seldom true" for the average acceptance item. The mean for the subscales of activity engagement and pain willingness were 23.0 (SD 14.2, range 0-59) and 18.1 (SD 9.7, range 0-53), respectively. The scores for other instruments are summarised in Table 1.

**Table 1:** Mean and SD of Scores of the Spanish Versions of Various Instruments (N = 205)

Instruments	Mean	SD
CPAQ (0-120)	40.9	18.5
Activity engagement subscale (0-66)	23.0	14.2
Pain willingness subscale (0-54)	18.1	9.7
PVAS (0-10)	7.9	1.5
HADS-anx (0-21)	12.2	4.3
HADS-dep (0-21)	11.2	4.7
PCS-total (0-52)	32.4	12.8
FIQ (0-100)	72.0	16.4
SF36-PF (0-100)	34.1	21.4
SF36-RP (0-100)	7.4	21.4
SF36-BP (0-100)	19.2	16.2
SF36-GH (0-100)	24.8	14.3
SF36-VT (0-100)	15.6	15.1
SF36-SF (0-100)	35.2	24.2
SF36-RE (0-100)	24.0	38.9
SF36-MH (0-100)	38.3	20.2

There was no significant association between CPAQ total score and most demographic characteristics, including age, sex, marital status, duration of pain or education level. However, work status (r = 0.140, P = 0.056) was almost correlated to CPAQ, suggesting that there might be an association (Table 2).

**Table 2:** Associations between the Spanish version of the CPAQ and demographic parameters.

Demographic parameters	Association	Significance
Age	0.025	*P = 0.736*
Sex	0.103	*P = 0.160*
Marital Status	0.186	*P = 0.321*
Education level	0.162	*P = 0.422*
Duration of pain	-0.042	*P = 0.591*
Work status	0.140	*P = 0.056*

For assessing face validity a sample of patients (N = 200) randomly recruited from the Spanish Association of Fibromyalgia were asked whether they thought that the test could adequately measure their pain acceptance. A total of 93.5% (187 out of 200) of them agreed.

**Figure 1:**
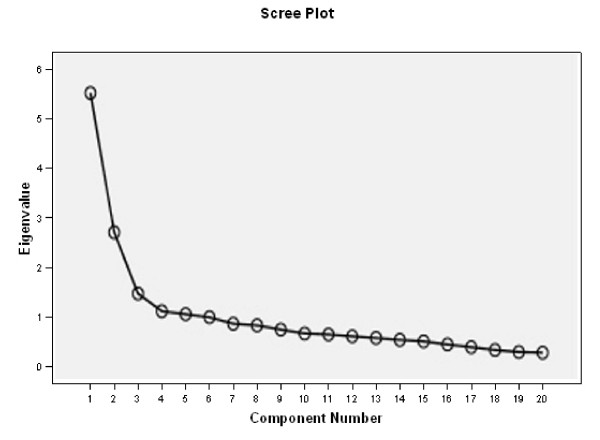
**Scree Plot indicates an optimal two-factor solution for the Spanish version of CPAQ**.

The overall ICC value was 0.83 with individual values (Table 3) ranging from 0.32 (item 20) to 0.88 (item 2). Regarding the two subscales of the CPAQ, test-retest reliability values are as follows: Activity engagement (ICC: 0.85; 95% CI: 0.81-0.89) and Pain willingness (ICC: 0.82; 95%CI: 0.79-0.86). Cronbach's α for the CPAQ was 0.83. The item-total correlations for most items were moderate (mean 0.406, SD 0.213). Communalities ranged from 0.169 (item 7) to 0.633 (item 1). The Scree Plot (Figure 1) indicated that a two-factor solution was optimal. Both factors had eigenvalues greater than one. Principal components analysis with Varimax Rotation revealed a satisfactory percentage of Total Variance explained by the two factors 27.4% and 13.4%, respectively (Table 4), as well as a corresponding Component Matrix (Table 5). These values are consistent with the original model of McCracken et al. [5] and subsequent studies [11,17], providing further support for the two-factor CPAQ.

**Table 3:** Item Means and SD, Intraclass Correlations (ICC) with 95% Confidence Interval (CI), Item-total correlations, Cronbach's α if item deleted for Spanish version of CPAQ (N = 205)

Item no.	Mean	SD	ICC (95% CI)	Item-total correlation	Cronbach's α if item deleted
1	2.4	1.9	0.83 (0.73-0.90)	0.592	0.81
2	2.1	1.7	0.88 (0.80-0.92)	0.583	0.81
3	1.7	1.8	0.72 (0.55-0.83)	0.480	0.82
4	1.9	1.8	0.56 (0.27-0.73)	0.367	0.82
5	1.9	1.9	0.54 (0.25-0.72)	0.415	0.82
6	2.1	1.8	0.82 (0.72-0.89)	0.566	0.81
7	2.5	2.0	0.61 (0.36-0.76)	0.164	0.83
8	2.2	1.8	0.75 (0.60-0.85)	0.488	0.81
9	1.7	1.7	0.85 (0.76-0.91)	0.554	0.81
10	2.1	1.9	0.64 (0.41-0.78)	0.495	0.81
11	2.5	2.1	0.81 (0.69-0.88)	0.241	0.83
12	2.4	1.9	0.65 (0.42-0.79)	0.395	0.82
13	2.1	2.0	0.47 (0.12-0.67)	0.197	0.83
14	2.1	1.9	0.52 (0.22-0.71)	0.312	0.82
15	1.9	1.8	0.80 (0.67-0.88)	0.531	0.81
16	2.3	2.0	0.65 (0.42-0.78)	0.126	0.83
17	1.7	1.8	0.60 (0.34-0.75)	0.401	0.82
18	1.6	1.9	0.59 (0.32-0.75)	0.378	0.82
19	2.0	1.9	0.69 (0.49-0.81)	0.509	0.81
20	1.3	1.7	0.32 (0.12-0.59)	0.339	0.82

Overall ICC was 0.83 (95% confidence interval: 0.79-0.86)

Cronbach's α of the total score was 0.83

**Table 4:** Forced two-factor solution by Principal Items Loading and Varimax Rotation for the Spanish version of the CPAQ (N = 205).

	Initial Eigenvalues			Extraction Sums of Squared Loadings			Rotation Sums of Squared Loadings		
	
Component	Total	% of Variance	Cumulative %	Total	% of Variance	Cumulative %	Total	% of Variance	Cumulative %
1	5.480	27.400	27.400	5.480	27.400	27.400	5.284	26.421	26.421
2	2.675	13.377	40.778	2.675	13.377	40.778	2.871	14.356	40.778
3	1.441	7.205	47.983						
4	1.086	5.429	53.412						
5	1.026	5.131	58.543						
6	.965	4.827	63.370						
7	.834	4.170	67.540						
8	.802	4.010	71.550						
9	.716	3.581	75.131						
10	.638	3.191	78.322						
11	.619	3.096	81.418						
12	.580	2.898	84.317						
13	.547	2.735	87.051						
14	.509	2.547	89.598						
15	.478	2.392	91.990						
16	.417	2.084	94.075						
17	.362	1.809	95.884						
18	.307	1.533	97.416						
19	.265	1.324	9.740						
20	.252	1.260	100.000						

**Table 5:** Two-factor solution: Factor Loadings by Principal Components Analysis on Items of the Spanish version of the CPAQ (N = 205).

	Factor Loadings		Communalities
**Summary Item Content**	**1**	**2**	

Q1 = I am getting on with the business of living no matter what my level of pain is	**.796**	.006	.633
Q2 = My life is going well, even though I have chronic pain	**.747**	.076	.564
Q6 = Although things have changed, I am living a normal life despite my chronic pain	**.739**	.057	.549
Q8 = There are many activities I do when I feel pain	**.706**	-.022	.499
Q9 = I lead a full life even though I have chronic pain	**.702**	.125	.508
Q19 = It's a relief to realise that I don't have to change my pain to get on with my life	**.686**	.043	.473
Q15 = When my pain increases, I can still take care of my responsibilities	**.674**	.101	.464
Q3 = It's OK to experience pain	**636**	.088	.412
Q10 = Controlling pain is less important than other goals in my life	**.619**	.108	.394
Q12 = Despite the pain, I am now sticking to a certain course in my life	**.593**	-.061	.355
Q5 = It's not necessary for me to control my pain in order to handle my life well	**.560**	.042	.316
Q20 = I have to struggle to do things when I have pain	.095	**.664**	.450
Q18 = My worries and fears about what pain will do to me are true	.140	**.662**	.458
Q14 = Before I can make any serious plans, I have to get some control over my pain	.062	**.620**	.388
Q13 = Keeping my pain level under control takes first priority whenever I'm doing something	-.041	**.549**	.303
Q11 = My thoughts and feelings about pain must change before I can take important steps in my life	.011	**.533**	.284
Q16 = I will have better control over my life if I can control my negative thoughts about pain	-.150	**.528**	.302
Q17 = I avoid putting myself in situations where my pain might increase	.269	**.528**	.351
Q4 = I would gladly sacrifice important things in my life to control this pain better	.218	**.483**	.281
Q7 = I need to concentrate on getting rid of my pain	-.032	**.410**	.169

(Items sorted according to loadings by factor and size for easier comprehension.)

The bold numbers (items) belong to the respective factor.

The Pearson correlation was used to assess the relationship between CPAQ and other psychometric instruments, and the results are summarised in Table 6. The CPAQ total score and the subscale for activity engagement were significantly correlated with all of the other psychometric instruments, including the VAS, HADS, PCS, FIQ and SF36. Whereas the subscale for pain willingness was only significantly correlated to certain scales.

**Table 6:** Correlation between Spanish version of CPAQ scores (total and subscales) and other Spanish instruments.

Instruments	Correlation (T)	Correlation (AE)	Correlation (PW)
VAS	-0.446 **	-0.427 **	-0.306 **
HADS-anx	-0.447 **	-0.456 **	-0.210 **
HADS-dep	-0.503 **	-0.605 **	-0.093
PCS-total	-0.461 **	-0.388 **	-0.344 **
FIQ	-0.603 **	-0.649 **	-0.231**
SF36-PF	0.397 **	0.415 **	0.169*
SF36-RP	0.248 **	0.298 **	0.056
SF36-BP	0.394 **	0.495 **	0.068
SF36-GH	0.438 **	0.410 **	0.239**
SF36-VT	0.395 **	0.381 **	0.197**
SF36-SF	0.450 **	0.526 **	0.101
SF36-RE	0.388 **	0.386 **	0.187**
SF36-MH	0.390 **	0.415 **	0.098

* Significant: P < 0.05

** Significant: P < 0.01

T = Total score of Spanish version of CPAQ

AE = Activity Engagement subscale of Spanish version of CPAQ

PW = Pain Willingness subscale of Spanish version of CPAQ

## Discussion

The psychometric properties of the Spanish version of the CPAQ among patients with fibromyalgia patients are adequate. The Scree plot indicated a two-factor construct of the translated questionnaire similar to its original English version. Both factors had eigenvalues greater than one. Principal Components with Varimax Rotation revealed a satisfactory percentage of Total Variance explained (40.7%) by the two factors. Looking at the Component Matrix of the two-factor construct, individual items could be allocated to the same subscales as they were in the English version of the CPAQ. Therefore, construct validity of the translated CPAQ can be supported.

We have selected a two-factor solution, although it was not the only possible solution. More than two factors had eigenvalues above 1, and the Scree plot was not absolutely clear in supporting this decision. We have selected this solution because it seems the more coherent from a clinical point of view. This is the same factor structure obtained by both the original authors [5] and the German and Cantonese validations previously carried out [16,17]. This has been defended by many other studies on pain acceptance [29-33]. We are currently carrying out a confirmatory factor analysis in a different population of patients with fibromyalgia, and preliminary results also seem to support this two-factor solution.

The Spanish version of the CPAQ showed good test-retest reliability (overall ICC 0.83 with 95% CI 0.79-0.86) and internal consistency reliability (Cronbach's α 0.83). Items n° 13 and 20 showed lower test-retest reliability than the other items (ICC < 0.5). In the original McCraken study, these data are not available [5], and in the Chinese validation study [17], ICC values are higher than 0.5 (item 13: 0.55 and item 20: 0.76). Both items belong to the subscale "Acceptance of pain". We are not sure why the test-retest reliability was low, but we suggest that cultural factors may play a role. Many Spanish pain patients have a quite passive viewpoint of pain and consider pain difficult to control by will power alone.

As tends to happen in fibromyalgia surveys, the SF-36 scores were below average. In this case, the average total score for CPAQ in this fibromyalgia group (mean 40.9 with SD of 18.5) was lower than other samples, where usually the mean is around 50 [12,13,16,17]. Statistical analysis showed that greater acceptance of pain and activity participation were associated with lower reported pain intensity, less anxiety, depression and emotional distress, as well as worse general health status and health-related quality of life (measured with the SF-36). These findings were in concordance with reports from previous studies [5,12,13]. It is also remarkable that the FIQ, a questionnaire specifically designed for fibromyalgia patients to measure health status, showed the highest correlation with the CPAQ, indicating how important acceptance is in predicting the impact of fibromyalgia. As far as we know, there are no acceptance studies among fibromyalgia patients using these scales, thus, it was not possible to compare our results.

Regarding demographic data, the variable duration of pain has received special attention, as it may indicate that acceptance of chronic pain is in some way a product of experience or something acquired over time. In fact, one recent work showed a positive correlation between the CPAQ and duration of pain [17]. However, in our research, as in the majority of studies, no correlation with duration of pain was found, suggesting that the length of time a person has suffered from pain may not account for whether a person is accepting of pain or not. Further studies may be required to clarify the factors contributing to such discrepancies.

These study results concurred with our prediction and supported the content validity of the Spanish version of the CPAQ. In the future, the Spanish CPAQ could help to illustrate treatment mechanisms. To reach this goal, the next step would be to assess the responsiveness of the CPAQ to intervention. Further research with longitudinal designs and multivariate models would be required to investigate treatment mechanisms.

As McCracken has already pointed out [5], the results of our study are limited because correlation methods cannot unambiguously determine whether acceptance leads to decreased levels of disability and distress or vice versa. Given the consistent relationship between acceptance and these measures, however, we would suggest that there are important behavioural processes at work. Experimental, longitudinal or clinical methods are needed to illuminate these processes. Finally, another possible limitation could be that the sample was recruited from a specialised clinic and, thus, may not be representative of all patients with fibromyalgia. This could be the reason for the lower CPAQ scores in this sample.

These findings hold potentially significant implications for the treatment of patients with fibromyalgia and chronic pain at a time in psychology when the usefulness of traditional, control-based approaches is under question. The increasingly popular Contextual Therapies approach proposes that attempting to control negatively valenced internal events, such as pain sensations and negative emotional reactions, is problematic. For example, from the Acceptance and Commitment Therapy (ACT) perspective, attempts to control aversive experiences are, in the best case, an unproductive endeavour that can hinder the pursuit of valued experiences or, in the worst case, an additional source of distress [34]. Experimental data suggest that some common control-based strategies to manage acute pain may be detrimental to functioning and adaptation [35,36]. Existing psychological treatments for chronic pain, such as ACT [37] or specific contextual therapy for chronic pain [38], aim to increase pain patients' pain acceptance on multiple levels.

Finally, in order to prevent misunderstandings, it should be noted that acceptance of chronic pain is but one part of a contextual model of chronic pain and its treatment. Other relevant processes include, among others, present-focused awareness, values-based guidance of actions and cognitive defusion. It will be interesting to continue to explore the influence of these processes on patient functioning.

In conclusion, the study confirms the adequate psychometric properties of the Spanish version of the CPAQ in fibromyalgia patients. Although acceptance is considered to be one of the key processes of recovery in pain syndromes, there have been hardly any studies in our country to enhance our knowledge of this concept. This study will make it easier to assess acceptance in Spanish populations.

## Competing interests

The authors declare that they have no competing interests.

## Authors' contributions

BR, JGC, BC and ASS conceived the study design. BC performed the clinical diagnosis of fibromyalgia. YLdH and BR collected the data. BR and JVL conducted the statistical analysis, and all authors interpreted the results, drafted the manuscript and read and approved the final manuscript.

## Supplementary Material

Additional file 1 Spanish version of the CPAQ. Click here for file
