# Supplementary material for: Validation of the Spanish version of the Chronic Pain Acceptance Questionnaire (CPAQ) for the assessment of acceptance in fibromyalgia
Source: Health Qual Life Outcomes. 2010 Apr 12;8:37. doi: 10.1186/1477-7525-8-37 (PMC2876109; doi:10.1186/1477-7525-8-37)
Supplement: Additional file 1 — Spanish version of the CPAQ. [file 1477-7525-8-37-S1.DOC]

Appendix I. Spanish version of the CPAQ

Instrucciones: A continuación, encontrará una lista de afirmaciones. Puntúe cada una de ellas haciendo un círculo en el número que mejor defina la frecuencia con la que dicha información es cierta para usted. Utilice la siguiente escala de puntuación para hacer su elección. Por ejemplo, si cree que una afirmación es “siempre cierta” para usted, deberá seleccionar un 6 en el espacio para su respuesta.

| 0 | 1 | 2 | 3 | 4 | 5 | 6 |
| --- | --- | --- | --- | --- | --- | --- |
| Nunca cierta | Muy raramente cierta | Raramente cierta | A veces cierta | A menudo cierta | Casi siempre cierta | Siempre cierta |

1. Continúo haciendo las cosas de la vida diaria sea cual sea mi nivel de dolor …….
2. Mi vida va bien, aunque tenga dolor crónico ……
3. No importa sentir dolor ……
4. Si tuviera que sacrificar cosas importantes de mi vida para controlar mejor este dolor, lo haría con mucho gusto ……
5. No necesito controlar el dolor para poder llevar bien mi vida ……
6. Aunque las cosas han cambiado, llevo una vida normal pese a mi dolor crónico ……
7. Tengo que concentrarme para poder librarme del dolor ……
8. Hay muchas actividades que hago cuando tengo dolor ……
9. Llevo una vida plena aunque tenga dolor crónico ……
10. Controlar el dolor es un objetivo menos importante que otros objetivos de mi vida ……
11. Antes de poder tomar decisiones importantes en mi vida, mis ideas y sentimientos hacia el dolor deben cambiar ……
12. A pesar del dolor, ahora me ciño a una dirección concreta en mi vida ……
13. Tener controlado el nivel de dolor es la primera prioridad cuando hago algo ……
14. Antes de poder hacer planes en serio, tengo que tener algo de control sobre el dolor ……
15. Cuando aumenta el dolor, puedo seguir ocupándome de mis responsabilidades ……
16. Podría controlar mejor mi vida si pudiera controlar mis pensamientos negativos sobre el dolor ……
17. Evito enfrentarme a situaciones en las que el dolor pudiera aumentar ……
18. Mis preocupaciones y miedos sobre lo que el dolor puede hacerme son auténticos ……
19. Es un alivio darse cuenta de que no tengo por qué cambiar el dolor para seguir con mi vida ……
20. Cuando tengo dolores, me cuesta mucho hacer cosas ……

**Puntuación:**

**Disposición para las actividades** = Sume los ítems 1, 2, 3, 5, 6, 8, 9, 10, 12, 15, 19.

**Aceptación del dolor** = Invierta las puntuaciones de los ítems 4, 7, 11, 13, 14, 16, 17, 18, 20 y súmelas.

**Tota**l = Disposición para las actividades + Aceptación del dolor.
